# The Process of Soil Carbon Sequestration in Different Ecological Zones of Qingtu Lake in the Arid–Semi-Arid Region of Western China

**DOI:** 10.3390/microorganisms12112122

**Published:** 2024-10-23

**Authors:** Tao Wang, Shengyin Zhang, Shuncun Zhang, Ming Shao, Zhaoyun Ding, Yanfang Zhou, Cuicui Su

**Affiliations:** 1 Northwest Institute of Eco-Environment and Resources, Chinese Academy of Sciences, Lanzhou 730000, China; wangtao192@mails.ucas.ac.cn (T.W.); shaoming@nieer.ac.cn (M.S.); 2 Gansu Academy of Agri-Engineering Technology, Wuwei 733006, China

**Keywords:** soil organic carbon, soil inorganic carbon, microbial diversity, mineral composition, arid and semi-arid regions

## Abstract

As a vital component of the global carbon pool, soils in arid and semi-arid regions play a significant role in carbon sequestration. In the context of global warming, increasing temperatures and moisture levels promote the transformation of barren land into wetlands, enhancing carbon sinks. However, the overdevelopment of oases and excessive extraction of groundwater lead to the opposite effect, reducing carbon sequestration. This study examines two soil types—meadow soil (MS) and swamp soil (SS)—from Qingtu Lake, an arid lake in western China. It analyzes the sources of soil inorganic carbon, the composition and origin of dissolved organic matter (DOM), and the relationships between microbes, soil organic carbon (SOC), soil inorganic carbon (SIC), mineral composition, and soil texture. The results indicate that inorganic carbon in the study area consists of both primary carbonate minerals and secondary pedogenic carbonates. The DOM primarily consists of two components, both identified as terrestrial humic substances. In meadow soils, bacterial activity drives the weathering of plagioclase, which releases Ca^2+^ necessary for the formation of pedogenic carbonates. Plagioclase also provides colonization sites for microbes and, along with microbial activity, participates in the soil carbon cycle. Within the soil community, bacteria appear to play a more critical role than fungi. In contrast, microbial contributions to the carbon cycle in swamp soils are weaker, with minerals predominantly interacting with organic carbon to form mineral-associated organic matter, thus promoting the soil carbon cycle. These findings have important implications for understanding soil carbon sinks under different micro-ecological conditions in arid and semi-arid regions. Through targeted human intervention, it is possible to enhance carbon sequestration in these areas, contributing to the mitigation of global climate change.

## 1. Introduction

As the most significant greenhouse gas, carbon dioxide (CO_2_) accounts for approximately 60% of the global greenhouse effect. With the advancement of atmosphere–soil carbon cycle theory, the role of soil in the carbon cycle has gradually gained attention. Soil represents the largest terrestrial carbon reservoir, containing roughly 2047 Pg of organic carbon and 1558 Pg of inorganic carbon [1]. The total soil carbon storage is approximately four times the carbon content in the atmosphere (750 Pg) [2] and six times that in vegetation (560 Pg) [3]. Dryland soils constitute about 52% of the global soil carbon pool [1,4], encompassing 32% of the global soil organic carbon (SOC) pool and 80% of the global soil inorganic carbon (SIC) pool. In the context of climate change and increasing global aridification, drylands are expanding, leading to land degradation and desertification [5,6,7]. Soil carbon in arid regions is highly sensitive to these drought-driven environmental and mechanistic changes and plays a substantial role in the overall loss of the global soil carbon pool [8,9,10]. Previous research has demonstrated that increased aridity leads to a reduction in soil organic carbon content [11,12,13,14]. However, increased aridity (or decreased precipitation) often coincides with an increase in SIC content [15,16,17].

In arid regions, soil acts as a medium for fixing atmospheric CO_2_, leading to the formation of secondary carbonates. These secondary carbonates initially exist in the soil solution as dissolved inorganic carbon (DIC). As the soil solution evaporates, DIC may combine with Ca^2+^ in the solution, transforming into solid-phase carbonate minerals, or it may infiltrate into the groundwater system, remaining in liquid form in relatively enclosed saline basins [18,19,20]. Based on the source of their carbonate ions, soil carbonates can be classified into diagenetic carbonates (or primary carbonates) and pedogenic carbonates (or secondary carbonates). Diagenetic carbonates result from physical weathering and mechanical transportation without involving carbon sequestration, but may serve as an ion source for pedogenic carbonates in the carbon sequestration process [21]. Pedogenic carbonates are formed by atmospheric CO_2_ dissolving in the soil solution to form carbonate or bicarbonate ions, which then precipitate with Ca^2+^ and Mg^2+^ under suitable moisture and pH conditions [22,23]. The CO_2_ dissolved in forming pedogenic carbonates originates directly or indirectly from the atmosphere, making the formation of secondary carbonates the primary carbon sequestration process [24]. Carbon isotopes have been successfully used to distinguish between diagenetic and pedogenic carbonates [25,26,27]. The δ^13^C values of pedogenic carbonates typically range from −10 to 0‰, while those of primary carbonates are around 0‰, approximately −2‰ to +2‰ [28,29,30]. Studies have shown that different mineral types can also help quantitatively distinguish between diagenetic and pedogenic carbonates [31,32].

SOC can be subdivided into dissolved organic carbon (DOC), particulate organic carbon (POC), and mineral-associated organic carbon (MAOC). POC has a relatively short mean residence time [33]. Unless located in environments where decomposition is limited by physical or physiological constraints on microbial activity [34], POC tends to accumulate in locations where decomposition is present [35]. In contrast, MAOC is formed through the adsorption of microbial residues, decomposition products, and soluble plant inputs onto soil mineral surfaces [36,37,38]. SOC and POC can undergo exchange through biogeochemical transformations, adsorption, desorption, aggregation, and dissolution processes [39,40,41,42,43]. DOC represents the most dynamic and bioavailable fraction of SOC, playing a critical role in biogeochemical cycles [44,45,46]. DOC can become part of the mineral-associated SOC pool by binding to fine soil particles [47]. Additionally, DOC can stimulate soil microbial activity and enhance organic matter decomposition [48]. In summary, OC provides carbon substrates and energy sources for sedimentary microorganisms, while these microorganisms drive benthic biogeochemical processes, thereby altering and influencing the quantity and quality of DOC [49,50,51]. Therefore, the impact of microorganisms on SOC and SIC should not be underestimated. Moreover, microbial biomass varies across different land types, and the combined effects of microorganisms and minerals on SOC also differ [52,53].

Approximately 7% of the world’s land is threatened by salinization, and the expanding area of saline-alkaline soils makes the study of carbon sinks in these soils increasingly important [54,55]. Soil salinization is more prevalent in the arid regions of north-west China. Qingtu Lake, an inland arid lake in Gansu Province, is located between the Badain Jaran and Tengger Deserts, and serves as the terminal lake of the Shiyang River. The lake’s water supply primarily comes from the Hongyashan Reservoir. Due to periodic artificial water replenishment, silicate input, and alternating warm–wet and cold–dry conditions in the arid region, Qingtu Lake experiences significant accumulation of both organic and inorganic carbon, especially during the autumn–winter ecological water replenishment and spring–summer natural evaporation periods. Microbial processes play a crucial role in mediating organic and inorganic interactions, making Qingtu Lake a representative site and a valuable window for studying carbon sinks in arid regions. Following ecological water replenishment, the vegetation diversity in the study area increased significantly. However, existing research lacks a comprehensive analysis of the sources and transformations of organic and inorganic carbon across different ecological types. To better reflect the impact of climate change on soils while avoiding diagenetic effects, this study collected surface soil samples (0–10 cm) from Qingtu Lake, focusing on meadow soil (MS) and swale soil (SS). The research primarily involved the following aspects: (1) Isotope analysis techniques were used to determine carbonate carbon isotopes, and X-ray diffraction (XRD) was employed to analyze soil mineral composition, exploring the sources of inorganic carbon; (2) UV-visible absorbance spectroscopy (SUVA) and three-dimensional fluorescence excitation-emission matrix (EEM) spectroscopy were used to analyze the composition and sources of DOC; and (3) The relationships between organic carbon, inorganic carbon, minerals, and microbes in Qingtu Lake were investigated by integrating microbial data, soil mineralogy, and soil texture characteristics.

## 2. Materials and Methods

### 2.1. Study Area

The Shiyang River Basin is located in the eastern part of the Qilian Mountains, with geographical coordinates approximately between 100°57′–104°57′ E and 37°02′–39°17′ N. The basin is about 300 km long, with a total area of 41,600 square kilometers. According to geographical zoning, the Shiyang River Basin is situated in the transitional zone between the monsoon region and the arid region. The modern climate of this area is influenced by both the Asian monsoon and the westerlies. Qingtu Lake, located in the Minqin Basin, is the terminal lake of the Shiyang River Basin. It falls within the northern warm and arid zone, one of the three climatic zones along the Shiyang River. The area has an elevation of 1300 to 1500 m, an annual precipitation of 50–200 mm, and an annual evaporation rate of 2000–2600 mm. Desert vegetation is widely distributed in this region. The main regional vegetation is *Phragmites australis*, *Kalidium foliatum*, *Suaeda glauca*, *Haloxylon ammodendron*, etc.

### 2.2. Site Description and Soil Sampling

**Figure 1:**
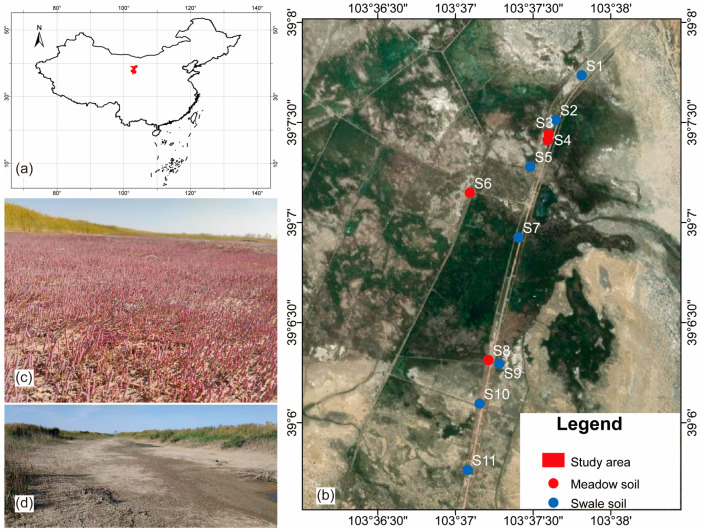
The study area is located in Wuwei City, Gansu Province, China (**a**), and the distribution of sampling sites is shown in (**b**), with soil types of meadow soil (the dominant vegetation type is *Kalidium foliatum*) (**c**) and swale soil (**d**). S1–S11: soil samples from 11 sites in Qingtu Lake, with 7 sites for SS (swale soil) samples and 4 sites for MS (meadow soil) samples.

Sampling points were arranged from upstream to downstream, extending from the lake delta towards the Tengger/Badan Jilin deserts. The locations were selected in areas with minimal human disturbance and convenient access. Additionally, two research plots were chosen based on different soil types to ensure a comprehensive analysis. In September 2023, soil samples were collected from 11 sites in Qingtu Lake, with 7 sites for SS (swale soil) samples and 4 sites for MS (meadow soil) samples (Figure 1). Surface soil samples (0–10 cm) were collected from each site using a soil corer with a diameter of 5 cm and a length of 10 cm. Three subsamples from each site were combined into a composite sample, which was then sieved (2 mm), placed in sterile microbial sampling bags, and transported to the laboratory in insulated containers kept below −10 °C. Samples designated for DOM component analysis were freeze-dried, while those for microbial diversity analysis were stored at −80 °C until shipped to a specialized testing facility (stored for a total of three days). Samples for other experiments were air-dried at room temperature. All experiments at each sampling site were performed in triplicate to ensure accuracy and reproducibility.

### 2.3. Soil Edaphic Property Measurements

Soil pH was measured using a PB-10 digital pH meter (Sartorius, Göttingen, Germany) in a mixture of fully air-dried soil and deionized water at a ratio of 1:2.5. Soil electrical conductivity (EC) was measured using a DDS-307A conductivity meter (Precision & Scientific Instrument Co., Shanghai, China) in a 1:5 (*w*/*v*) soil-to-deionized water mixture, and soluble ions were tested using an ion chromatograph (ECO IC Metrohm, Gallen, Switzerland) and the ${CO}_{3}^{2-}$ and ${HCO}_{3}^{-}$ contents were determined according to a Chinese industrial standard (DZ/T 0064. 49-93) [56]. After removing carbonates by acidifying the samples with 7% hydrochloric acid and rinsing with deionized water until neutral, SOC content was determined using a CS-902G analyzer. Total carbon (TC) content in the soil was directly measured using a CS-902G analyzer, and SIC was calculated using the formula SIC = TC − SOC.

Soil texture (clay/silt/fine sand/coarse sand) was classified according to the international system using a particle size analyzer (Malvern Masterizer 2000, Worcestershire, UK) within a measurement range of 0.02–2000 μm after removing organic matter and carbonates from the samples with hydrogen peroxide and hydrochloric acid. Mineral composition was assessed using CuK radiation at 40 kV and 40 mA. The mineral content and percentage of the samples were calculated using MDI Jade 6 software. The calculation of mineral content followed the Chinese industrial standard SY/T 5163-2010 [57]. The δ^13^C analysis of carbonates was conducted using a gas stable isotope mass spectrometer (Thermo Scientific Delta V—GasBench II, Waltham, MA, USA). Before measurement, plant residues and microbial shells were removed. The samples were then ground to less than 200 mesh. The processed sediment samples were placed in glass vials and inserted into the aluminum heating block of GasBench II. The samples (0.1 g) were then purged with helium for 7 min and treated with supersaturated phosphoric acid (98%) for 2 h; the reaction temperature was 72 °C. Finally, the CO_2_ generated by the reaction was detected by Delta V, with helium as the carrier gas. NBS-18 was used as the international reference material, and GBW04405 was used as the national reference material.

### 2.4. Measurement of Soil Microbial Variables

Total DNA from the sediment samples (~0.5 g of wet sediment) was extracted using the FastDNA SPIN Kit for Soil (MP Biomedicals, Santa Ana, CA, USA). Bacteria were detected using the primer pair F (ACTCCTACGGGAGGCAGCA)/R (GGACTACHVGGGTWTCTAAT), and fungi were detected using the primer pair F (CTTGGTCATTTAGAGGAAGTAA)/R (GCTGCGTTCTTCATCGATGC) on a GeneAmp 9700 (ABI). The raw reads obtained from sequencing were first filtered using Trimmomatic v0.33 software, and then the primer sequences were identified and removed using cutadapt 1.9.1 software to obtain clean reads without primer sequences. Denoising was performed using the dada2 method [58] in QIIME2 2020.6 [59], and the paired-end sequences were merged and chimeric sequences removed to obtain the final effective data. Alpha diversity indices such as Chao1 and Shannon were calculated using QIIME2.

### 2.5. Water-Extractable DOM

Analyzing DOM constituents in soil using ultraviolet-visible absorption spectroscopy (SUVA) and three-dimensional fluorescence excitation-emission matrix spectroscopy is a well-established and widely adopted technique. The open-source computational workflow, the MATLAB R2023b software package and the DOM component comparison database provided strong support for the matching of soil DOM components in this study. A 1.0 g soil sample was weighed and placed in a 50 mL centrifuge tube, then mixed with ultrapure water in a soil-to-water ratio of 10:1. The mixture was continuously shaken at 180 r·min^−1^ for 24 h in a light-proof room at room temperature, then centrifuged at 4000 r·min^−1^ for 30 min. The DOM filtrate was obtained by filtering through a 0.45 μm cellulose nitrate membrane and stored in the dark at 4 °C. Spectral analysis was completed within 24 h to prevent potential biodegradation. The ultraviolet-visible absorbance values of the released DOM samples were analyzed using a UV-visible spectrophotometer (UV Probe-1780, Shimadzu, Kyoto City, Japan). Ultraviolet-visible absorbance spectra were measured for each extract sample in the wavelength range of 200 to 800 nm using ultrapure water as a blank and a 1 cm quartz cuvette. Fluorescence was measured using a fluorescence spectrophotometer (Agilent Cary Eclipse, Santa Clara, CA, USA) with a 1 cm quartz cuvette with four optical windows. Emission scans were performed from 230 nm to 550 nm in 5 nm increments, and excitation wavelengths ranged from 230 nm to 450 nm in 5 nm increments. The detector was set to high sensitivity, and the scanning speed was maintained at 24,000 nm/min. EEM data were corrected with a water blank. Before analysis, Raman and Rayleigh scattering were removed according to the procedure of Bahram et al. [60]. Parallel factor analysis was performed to decompose the excitation-emission matrix spectra using the DOMFluor toolbox (http://www.models.life.ku.dk/ (accessed on 6 July 2024)) [61]. The obtained model was matched using the OpenFluor database (https://openfluor.lablicate.com/ (accessed on 5 August 2024)) [62].

### 2.6. Statistical Analysis

Statistical analyses were performed using SPSS (version 26.0; IBM, Armonk, NY, USA). Spearman correlation analysis was conducted using the “Corrplot” R package, and Mantel tests were performed using the “linkET” R package. Data charts were plotted using Origin software (version 2021; OriginLab, Northampton, MA, USA).

We used linear regression functions to analyze the relationship between SOC, SIC, microorganisms, and minerals in different ecological zones, and used the Mantel test to test the correlation between SOC, SIC, microbial alpha diversity, major minerals, and soil texture in two ecological zones. The parallel factor analyses and contour plots of the DOM were performed using MATLAB R2023b (MathWorks, Natick, MA, USA).

## 3. Results

### 3.1. pH, EC, Water-Soluble Anions and Cations, Soil Texture, and Mineral Characteristics

**Figure 2:**
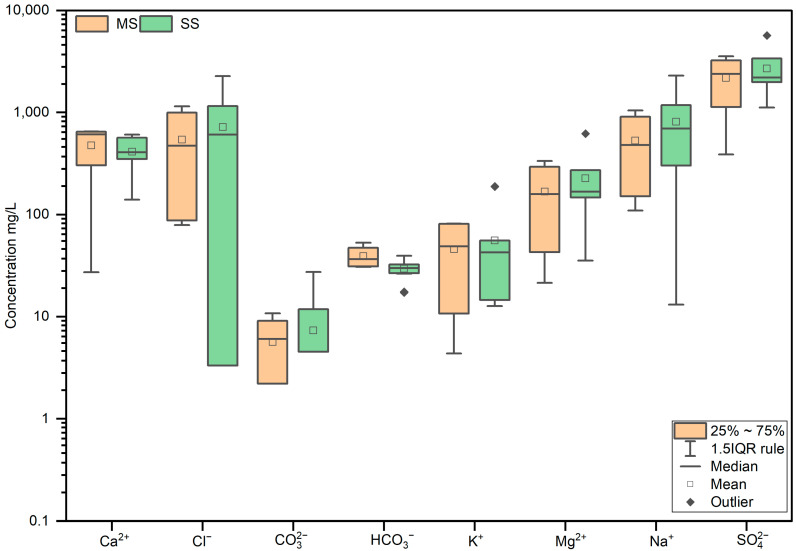
Concentrations of anions and cations in the soil samples of MS and SS in the Qingtu Lake.

The water-soluble ions differed slightly between the two study areas, with the SS showing a higher total concentration of soluble ions. The ion concentrations in the SS were ${SO}_{4}^{2-}$ (2699.3 mg/L) > Na^+^ (810.8 mg/L) > Cl^−^ (720.7 mg/L) > Ca^2+^ (412.2 mg/L) > Mg^2+^ (226.5 mg/L) > K^+^ (55.9 mg/L) > ${HCO}_{3}^{-}$ (29.5 mg/L) > ${CO}_{3}^{2-}$ (7.4 mg/L), while in the MS, the concentrations were ${SO}_{4}^{2-}$ (2181.5 mg/L) > Cl^−^ (542.5 mg/L) > Na^+^ (530.2 mg/L) > Ca^2+^ (475.3 mg/L) > Mg^2+^ (168.5 mg/L) > K^+^ (46.1 mg/L) > ${HCO}_{3}^{-}$ (39.3 mg/L) > ${CO}_{3}^{2-}$ (5.9 mg/L) (Figure 2 and Table S1).

The soil samples from both study areas were alkaline, with pH values of 8.8 and 9.0, respectively. The conductivity differed significantly between the two areas. The average conductivity in the SS was 6.04 ms/cm, while it was 3.80 ms/cm in the MS (Table S1). This study identified four soil texture categories: clay (<2 μm), silt (2–20 μm), fine sand (20–200 μm), and coarse sand (200–2000 μm). In the SS, clay content ranged from 0.4% to 2.8% (average 1.0%), silt from 14.5% to 61.6% (average 33.2%), fine sand from 35.6% to 80.3% (average 60.5%), and coarse sand from 0% to 17.9% (average 5.3%). In the MS, clay content ranged from 0% to 0.7% (average 0.4%), silt from 6.5% to 19.9% (average 13.7%), fine sand from 74.5% to 89.1% (average 81.1%), and coarse sand from 2.8% to 7.8% (average 4.8%). Compared to the SS, the MS had a higher fine sand content and lower silt content (Table S2).

Mineralogical analysis of the soil showed that both study areas had the same types of minerals, with minimal differences in mineral content. Quartz was the dominant mineral, with the following compositions: The mineral content distribution of the SS was quartz (11–44%, average 28%), plagioclase (11–42%, average 24%), potassium feldspar (0–36%, average 15%), calcite (6–19%, average 11%), gypsum (0–45%, average 12%), clay minerals (4–14%, average 7%), hematite (0–9%, average 3%), and ankerite (0–4%, average 1%); the distribution of mineral content in the MS was quartz (12–38%, average 28%), plagioclase (17–27%, average 22%), potassium feldspar (4–31%, average 13%), calcite (9–21%, average 15%), gypsum (0–29%, average 13%), clay minerals (2–5%, average 4%), hematite (1–9%, average 4%), and ankerite (0–4%, average 1%) (Table S3).

### 3.2. Microbial Community Structure Diversity

Both the SS and the MS had eight core prokaryotic bacterial genera and eight core fungal genera (Figure S1). In terms of prokaryotes, the core phyla in both areas were largely the same, with most belonging to Proteobacteria, Bacteroidota, Actinobacteriota, Chloroflexi, and Firmicutes, while the remaining phyla belonged to Gemmatimonadota, Acidobacteriota, Patescibacteria, and Desulfobacterota. Regarding fungi, the core phyla were also similar between the two areas, with most fungi belonging to Ascomycota, Basidiomycota, and Chytridiomycota, while the remaining fungi belonged to Mortierellomycota, Glomeromycota, Rozellomycota, and Mucoromycota (Figure S2). The Alpha diversity between the two study areas differed significantly; the bacterial Chao1 index was much higher in the MS (2235) than in the SS (1677), and the Shannon index was similarly higher in the MS (9.17) compared to the SS (8.7). The fungal Chao1 index was also higher in the MS (369.5) compared to the SS (211.5), but the fungal Shannon index showed little difference between the MS (5.92) and the SS (5.17) (Table S4).

### 3.3. SOC, SIC, DOM Characteristics, and Carbon-Oxygen Isotope Characteristics

**Figure 3:**
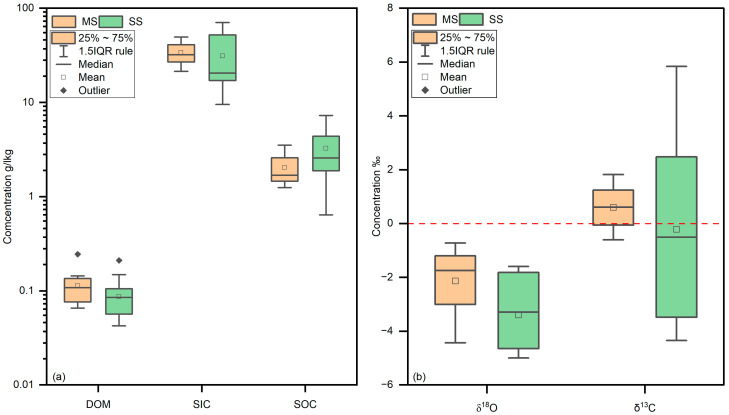
DOM, SIC, SOC, and δ^13^C, δ^18^O content. The DOM content in the SS ranged: the MS range from 0.07 mg/g to 0.25 mg/g (average 0.11 mg/g) (**a**) and ranged from −4.1‰ to −0.8‰ (average −2.1‰) (**b**).

The results of SOC, SIC, DOM content, and carbonate carbon and oxygen isotopes showed that SOC and SIC contents were similar between the two study areas, with SOC content ranging from 0.6 mg/g to 7.3 mg/g (average 2.8 mg/g). SIC content was much higher than SOC content (10.0 mg/g to 70.6 mg/g, average 32.3 mg/g), which aligns with the conclusions mentioned earlier. The DOM content in the SS ranged from 0.04 mg/g to 0.21 mg/g (average 0.09 mg/g), while in the MS, it ranged from 0.07 mg/g to 0.25 mg/g (average 0.11 mg/g) (Figure 3a). The carbonate carbon isotope δ^13^C content in the SS (−4.3‰ to 5.8‰, average −0.2‰) was lower than in the MS (−0.6‰ to 1.7‰, average 0.6‰), while the carbonate δ^18^O content was similar between the two study areas. In the SS, δ^18^O content ranged from −5.0‰ to −1.7‰ (average −3.4‰), and in the MS, it ranged from −4.1‰ to −0.8‰ (average −2.1‰) (Figure 3b).

### 3.4. Composition of DOM in Soil

**Figure 4:**
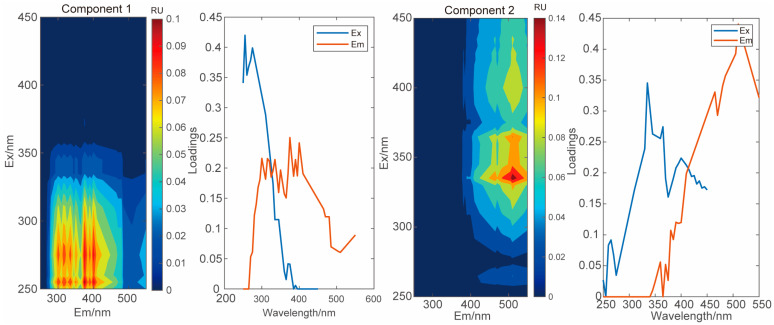
PARAFAC model identifying two DOM components (Comp 1, Comp 2).

We conducted UV-vis absorbance and 3D fluorescence analysis of DOM in both study areas as a whole. Based on EEMs-PARAFAC observations, the optical characteristics of sedimentary DOM could be divided into two DOM components (Figure 4, Comp 1 and 2), which were consistent with previous reports and/or fully matched the open-source fluorescence database (Open Fluor). Both compounds were humic substances, with component 1 likely associated with more biodegradable humic-like terrestrial substances [63,64]. Component 2 was likely fulvic acid-like substances in the soil [65,66].

### 3.5. Relationships Between SOC, SIC, Microorganisms, and Minerals in Soil

**Figure 5:**
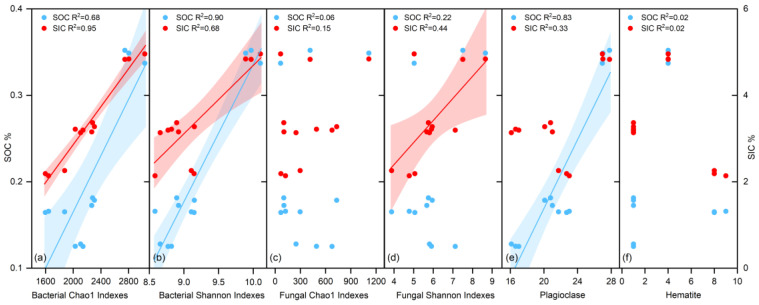
Relationships between SOC, SIC and microorganisms and minerals in MS. Red shadow indicates SOC in relation to microorganisms and minerals, blue shadow indicates SIC in relation to microorganisms and minerals. (**a**,**b**) indicate bacterial alpha diversity, (**c**,**d**) indicate fungal alpha diversity, (**e**,**f**) indicate major minerals.

**Figure 6:**
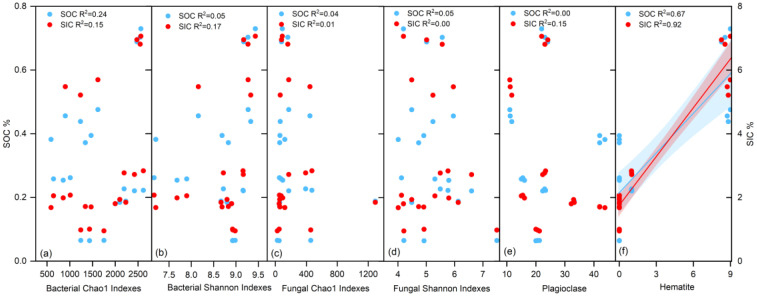
Relationships between SOC, SIC, microorganisms, and minerals in SS. Red shadow indicates SOC in relation to microorganisms and minerals, blue shadow indicates SIC in relation to microorganisms and minerals. (**a**,**b**) indicate bacterial alpha diversity, (**c**,**d**) indicate fungal alpha diversity, (**e**,**f**) indicate major minerals.

We separately analyzed the relationships between SOC, SIC, bacterial alpha diversity (Chao1 and Shannon), fungal alpha diversity (Chao1 and Shannon), and different minerals (mainly calcite, plagioclase, and hematite) in SS and MS. The results indicate: (1) In MS, the relationship between microbial alpha diversity and both SOC and SIC is more pronounced. The bacterial Chao1 index and Shannon index show strong positive correlations with SOC (R^2^ = 0.68, R^2^ = 0.95) and SIC (R^2^ = 0.90, R^2^ = 0.68) (Figure 5a,b). In contrast, the fungal Chao1 index does not exhibit significant positive correlations with either SOC or SIC (R^2^ = 0.06, R^2^ = 0.15, Figure 5c). The fungal Shannon index shows a positive correlation with SIC (R^2^ = 0.44), but not with SOC (R^2^ = 0.22, Figure 5d). Plagioclase demonstrates a strong positive correlation with SOC (R^2^ = 0.83), but a weaker relationship with SIC (R^2^ = 0.33, Figure 5e). Hematite shows no significant correlation with either SOC or SIC in MS (R^2^ = 0.02, R^2^ = 0.02, Figure 5f). (2) In SS, the correlation between microbial alpha diversity and both SOC and SIC is weaker, with almost no correlation observed (Figure 6a–d). Analysis of the relationships between SOC, SIC, and minerals reveals that SOC and SIC show no correlation with plagioclase (R^2^ = 0.00, R^2^ = 0.15, Figure 6e). However, there is a significant positive correlation between SOC, SIC, and hematite (R^2^ = 0.67, R^2^ = 0.92, Figure 6f).

## 4. Discussion

### 4.1. Influence of Soil Microbial Communities on SIC Formation and Analysis of SIC Sources

**Figure 7:**
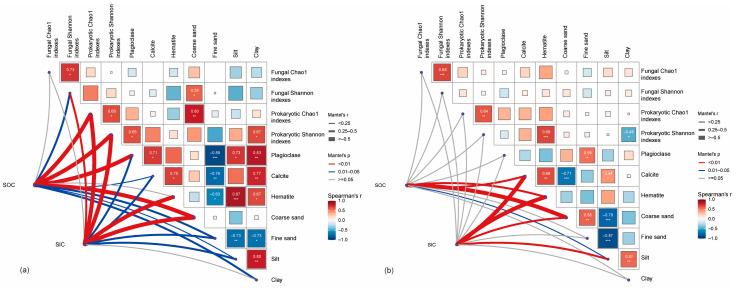
Pairwise comparisons of environmental factors in MS (**a**) and SS (**b**), colour gradients indicate Spearman’s correlation coefficients, edge widths correspond to the Mantel’s r statistic for the correlations, and edge colours indicate statistical significance. * *p* < 0.05, ** *p* < 0.01, *** *p* < 0.001.

Previous studies have suggested that SIC is mainly influenced by abiotic factors such as soil pH, soil moisture, CO_2_ partial pressure, and Ca^2+^ concentration [67,68]. However, our research findings indicate that the SIC content in MS increases with higher bacterial diversity, as measured by the Chao1 index, and bacterial richness, as measured by the Shannon index. This suggests that greater bacterial alpha diversity, compared to fungal diversity, is more conducive to SIC formation (Figure 5a–d) [69]. In contrast, the relationship between SIC and microbial activity is less pronounced in SS areas (Figure 6a–d). Specifically, higher bacterial alpha diversity is more favorable for the formation of calcite minerals in soil, and there is a positive correlation between bacterial alpha diversity and the presence of plagioclase [70,71]. This may be due to the bioweathering of plagioclase under bacterial influence, leading to the release of Ca^2+^, which subsequently combines with dissolved CO_2_ to form CaCO_3_, as one of the SIC sources (Figure 7a) [52,72,73].

The carbon isotope composition of soil carbonates is not inherited from mineral composition components but is instead determined by the carbon isotope values of soil CO_2_, which are influenced by root and microbial respiration, organic matter decomposition, and the proportion of atmospheric CO_2_ [74,75]. Additionally, in soils, dolomite is almost entirely derived from parent material and does not dissolve to reform [21]. Thus, the presence of dolomite indicates the presence of diagenetic carbonates [31,32]. The δ^13^C values in the study area range from −4.3‰ to 5.8‰, with an average value of 0.1‰. We also observed that some samples from both MS and SS contain only calcite, while others contain both calcite and dolomite (Table S2). Therefore, we infer that the carbonates in the study area include both pedogenic and diagenetic carbonates [76,77,78]. Based on these data, we conclude that the primary sources of SIC in Qingtu Lake are twofold: inorganic carbon from diagenetic carbonates derived from the transport and deposition of parent material, and inorganic carbon in pedogenic carbonates formed by the interaction of microorganisms (mainly bacteria), plagioclase, carbonate ions and bicarbonate ions in soil.

### 4.2. Relationships Among SOC, SIC, Microbes, and Minerals in Different Ecological Zones

**Figure 8:**
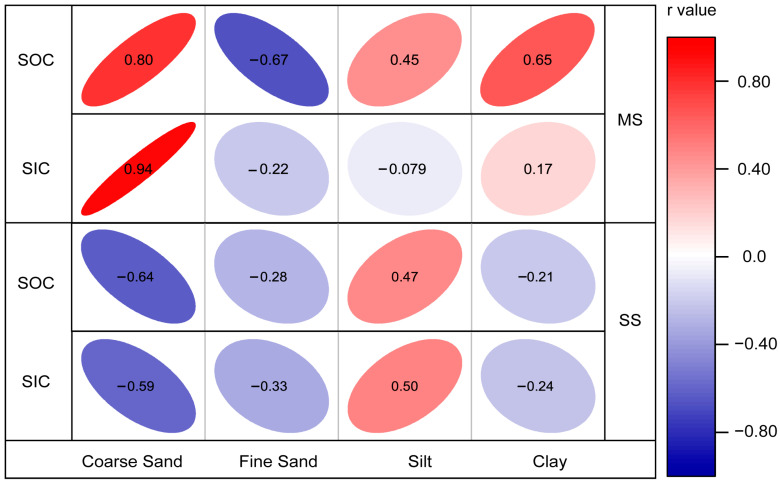
SOC, SIC, and soil texture Pearson correlation analyses between MS and SS.

The three-dimensional fluorescence analysis indicates that the DOC in the study area is composed of two primary components, both originating from terrestrial humic substances. Among these, component 2 is also influenced by microbial activity. Given that DOC accounts for only 5% of the SOC, and considering that SIC is the predominant form of carbon storage in the study area, it is crucial to focus on carbon forms beyond DOC. In MS, there is a strong positive correlation between SOC, SIC, and coarse sand content (Figure 8). Conversely, in SS, SOC and SIC exhibit a significant negative correlation with coarse sand content and a positive correlation with silt content. We propose that the higher microbial species diversity in MS facilitates the transformation of humic substances into SOC, while the presence of coarse sediment provides ample space for the preservation of both SOC and SIC [79,80]. Across different ecological zones, SOC and SIC demonstrate varying relationships with mineral types, soil texture, and microbial diversity. Mantel analysis reveals that in MS, microorganisms—particularly bacteria—play a significant role in influencing SOC and SIC. Higher bacterial biomass is conducive to the formation of calcite minerals, and plagioclase may offer colonization sites for microbes, potentially participating in microbial life processes [81]. This interaction results in a significant positive correlation between plagioclase content and the bacterial Shannon index in soils with lower clay mineral content. In contrast, fungal diversity appears to have a minimal impact on SOC and SIC (*p* > 0.05, Figure 7a).

The study found that SOC exists in multiple forms and can combine with minerals to form mineral-associated organic matter, such as water-soluble organic matter (WSOM), weakly-bound clusters, metal-bound complexes, and strongly bound clusters [82,83], which is consistent with our findings. The Mantel test of SOC and SIC in SS, along with mineral composition, bacterial alpha diversity, fungal alpha diversity, and soil physicochemical properties, indicates that organic and inorganic carbon in SS are essentially unaffected by microbial diversity, but are most significantly related to calcite and hematite (*p* < 0.01, Figure 7b). This suggests that SOC in SS may be stored in minerals in the form of Fe/Al-OM complexes [84] or protected by short-range-order Fe/Al-bearing minerals.

## 5. Conclusions

The study of the Qingtu Lake region reveals that SIC primarily originates from two sources. The first is inorganic carbon from diagenetic carbonate rocks, brought by the parent material of the soil. The second source is inorganic carbon from pedogenic carbonate, formed by atmospheric CO_2_ dissolved in water, which then combines with Ca^2+^ released through bacterial-mediated plagioclase dissolution, as well as Ca^2+^ and Mg^2+^ transported by water. The influence of microorganisms on SIC is mainly reflected in the biological activity of bacteria, which facilitate SIC formation by providing essential Ca^2+^ through the dissolution of plagioclase. DOM in the area is primarily derived from terrestrial humic substances, with microorganisms playing a role in its transformation process. The roles of minerals and microorganisms in the sequestration of SOC and SIC vary between different ecological zones. In MS, microorganisms, particularly bacteria, along with minerals like plagioclase and hematite, contribute positively to the sequestration of SOC and SIC. Minerals provide colonization sites for microorganisms, which, in turn, facilitate the conversion of humic substances into SOC. In contrast, microorganisms in SS have a lesser impact on the carbon pool, with SOC likely existing in the form of organic matter bound to different types of minerals. Following ecological water transfer, the vegetation diversity in the study area has significantly increased. This study focuses only on MS and SS, where the dominant vegetation species are *Kalidium foliatum* and *Phragmites*. Given that different vegetation types have varying effects on soil carbon sequestration, further studies on the carbon sequestration potential of arid and semi-arid lakes require a more refined classification of the microecosystem. Additionally, the comprehensive influence of microbial species, minerals, and other factors must be considered. Through appropriate human intervention, the carbon sequestration capacity of the study area can be enhanced, contributing to active efforts in mitigating global climate change.

## Data Availability

Data are contained within the article and Supplementary Materials.

## Supplementary Materials

The following supporting information can be downloaded at: https://www.mdpi.com/article/10.3390/microorganisms12112122/s1, Figure S1. Distribution of microbial genera; Figure S2. Characteristics of core microbial phyla; Table S1. Distribution of pH, EC (ms/cm), Water-Soluble Anions and Cations (mg/L) in SS and MS; Table S2. Soil texture characteristics in SS and MS; Table S3. Mineral composition characteristics of in SS and MS; Table S4. Microbial alpha diversity in SS and MS.
