# Supplementary material for: The Process of Soil Carbon Sequestration in Different Ecological Zones of Qingtu Lake in the Arid–Semi-Arid Region of Western China
Source: Microorganisms. 2024 Oct 23;12(11):2122. doi: 10.3390/microorganisms12112122 (PMC11596876; doi:10.3390/microorganisms12112122)
Supplement: Supplementary file 1 [file microorganisms-12-02122-s001.zip › microorganisms-3191223-supplementary.pdf]

The Process of Soil Carbon Sequestration in Different Ecological Zones of Qingtu Lake in the Arid–Semi-Arid Region of Western China

Tao Wang [1], Shengyin Zhang [1,*], Shuncun Zhang [1], Ming Shao [1], Zhaoyun Ding [2], Yanfang Zhou [2] and Cuicui Su [2]

[1] Northwest Institute of Eco-Environment and Resources, CAS, Lanzhou 730000, China; wangtao192@mails.ucas.ac.cn (T.W.); shaoming@nieer.ac.cn (M.S.)
[2] Gansu Academy of Agri-Engineering Technology, Wuwei 733006, China
* Correspondence: zhangshengyin@nieer.ac.cn

Table S1. Distribution of pH, EC, Water-Soluble Anions and Cations in SS and MS.

Soil types		Ca^{2+}	K^{+}	Mg^{2+}	Na^{+}	Cl^{-}	SO_4^{2-}	CO_3^{2-}	HCO_3^{-}	pH	EC
SS	min	140.19	12.67	35.45	13.10	0.00	1119.33	-0.26	17.26	8.16	1.63
	max	607.03	188.43	622.01	2295.37	2275.18	5672.95	27.42	39.47	9.43	13.25
	mean	412.10	55.85	226.40	810.66	720.63	2699.23	7.35	29.42	8.72	5.93
MS	min	27.21	4.37	21.40	109.74	78.81	388.92	-0.22	30.78	8.39	0.78
	max	651.49	81.55	334.37	1049.51	1148.58	3561.85	10.78	52.94	9.23	6.48
	mean	475.14	45.96	168.41	530.15	542.43	2181.43	5.66	39.21	8.88	3.70

Table S2. Soil texture characteristics in SS and MS.

Soil types	Value	coarse sand (%)	fine sand (%)	silt (%)	clay (%)
SS	min	0.00	34.78	14.21	0.38
	max	17.34	80.01	61.52	2.95
	mean	5.20	61.02	33.16	1.04
MS	min	2.80	73.92	6.54	0.04
	max	7.88	92.10	20.27	0.74
	mean	4.85	82.27	13.66	0.44

Table S3. Mineral composition characteristics of in SS and MS.

Soil types		Clay minerals (%)	Quartz (%)	Potassium feldspar (%)	Plagioclase (%)	Calcite (%)	Ankerite (%)	Gypsum (%)	Hematite (%)
SS	min	4	11	0	11	6	0	0	0
	max	14	45	37	44	20	4	47	9

	mean	7	28	15	24	11	1	12	3
MS	min	2	12	4	16	9	0	0	1
	max	5	41	32	28	22	4	30	9
	mean	3	29	13	22	15	1	13	4

Table S4. Microbial alpha diversity in SS and MS.

Soil types		Bacterial Alpha diversity		fungal Alpha diversity	
		Chao1	Shannon	Chao1	Shannon
SS	min	588.61	7.17	25.00	4.01
	max	2617.14	9.44	1292.07	7.49
	mean	1677.66	8.68	211.49	5.17
MS	min	1596.08	8.583	65.25	3.87
	max	3032.46	10.103	1113.43	8.65
	mean	2234.90	9.167	369.54	5.92

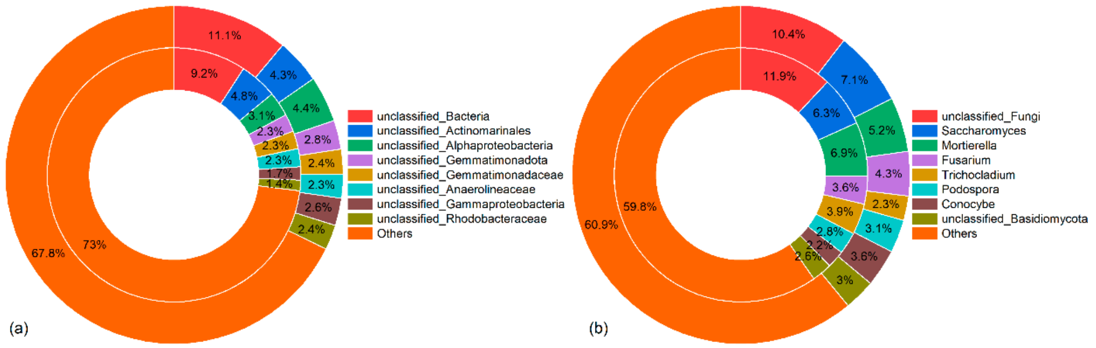

Figure S1. Distribution of microbial genera. a: The outer ring represents the distribution of bacteria in MS, while the inner ring represents the distribution of bacteria in SS; b: The outer ring illustrates the distribution of fungi in MS, and the inner ring illustrates the distribution of fungi in SS.

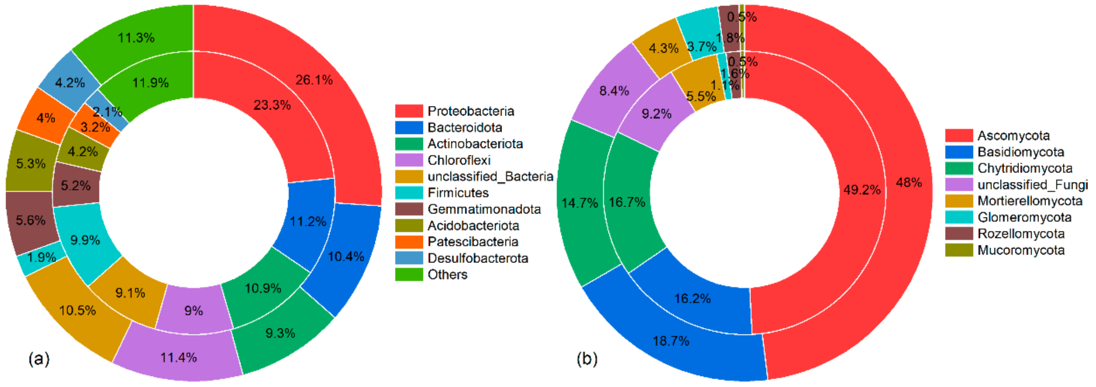

Figure S2. Characteristics of core microbial phyla. a: The outer ring represents the distribution of

core bacterial phyla in MS, and the inner ring represents the distribution of core bacterial phyla in SS. b: The outer ring represents the distribution of core fungal phyla in MS, and the inner ring represents the distribution of core fungal phyla in SS.
